# Disability-adjusted life years for respiratory syncytial virus in children under 2 years

**DOI:** 10.1186/s12889-020-09796-x

**Published:** 2020-11-10

**Authors:** Juana Patricia Sánchez Villamil, Fernando P. Polack, Jefferson Antonio Buendía

**Affiliations:** 1 Facultad de Ciencias Basicas, Universidad Antonio Nariño, Bucaramanga, Colombia; 2 Fundación Infant, Buenos Aires, Argentina; 3 Grupo de Investigación en farmacología y toxicología, Centro de Información y Estudio de Medicamentos y Tóxicos (CIEMTO), Facultad de Medicina, Universidad de Antioquia, Carrera 51D #62-29, Medellín, Colombia

**Keywords:** Global burden of disease, Colombia, Respiratory syncytial virus

## Abstract

**Background:**

Respiratory syncytial virus infection is the leading cause of bronchiolitis in Colombia. There is growing evidence about the impact of Respiratory syncytial virus on society in terms of years of life lost due to this condition. The objective of the present study is to determine the Disability-Adjusted Life Years for respiratory syncytial virus in children under 2 years in Colombia.

**Methods:**

Data from the national epidemiological surveillance system were used to estimate DALYs, calculated from the sum of years of life lost and years lived with disability due to RSV infection in Colombia. A bootstrapped method with 10,000 iterations was used to estimate each statistical parameter using the package DALY calculator in R.

**Results:**

In 2019, 260,873 years of life (CI95% 208,180–347,023) were lost due to RSV bronchiolitis in Colombian children under 2 years. The estimated rate was 20 DALYs / 1000 person-year (95% CI 16–27).

**Conclusion:**

This is the first report estimating the impact of RSV bronchiolitis morbidity and mortality in Colombia. The findings of the present study suggest that the actual burden and cost of bronchiolitis due to RSV is high. Prevention strategies, such as RSV vaccination, to reduce morbidity associated with RSV infection should be encouraged in our country.

## Background

Respiratory syncytial virus (RSV) is the most frequent cause of bronchiolitis worldwide. This virus causes 33.1 million episodes of RSV lower respiratory tract illness (LRTI), 3.2 million hospital admissions, and 59,600 in-hospital deaths in children younger than 5 years [1]. Not all patients have the same risk of death. Certain high-risk groups, including premature infants, infants with bronchopulmonary dysplasia, hemodynamically significant congenital heart disease, immunocompromised conditions, or severe neuromuscular disease are prone to experience severe RSV with high morbidity and mortality rates [2, 3]. The frequency or magnitude of association of these risk factors may change between different countries. The absence of seasonality, and the tropical climate, makes the behavior of RSV in Colombia differ from developed countries [4]. In this sense, having complete epidemiological information in tropical countries is necessary to design of health policies.

Mortality does not give an entire representation of the burden of disease produced by individuals in different populations. The burden of disease is evaluated using the disability-adjusted life year (DALY), a time-based measure that link years of life lost due to premature mortality (YLLs) and years of life lost due to time lived in states of less than full health, or years of healthy life lost due to disability (YLDs). One DALY means the loss of the equivalent of 1 year of full health [5]. With this measure we can be estimated diseases that do not cause death but do cause disability. The Global Burden of Diseases, Injuries, and Risk Factors (GBD) Study 2015 estimated that lower respiratory tract infections caused 2·74 million deaths and 103·0 million disability-adjusted life-years (DALYs) [6]. In this study DALYs, and deaths attributable to lower respiratory tract infections (LRTI). According to this study, in Colombia the DALYs attributable to lower respiratory tract infections in 2015 were 1.07 DALYs per 10,000 in all ages. Despite, that previous studies had estimated the burden of disease of LRTI, in term of years of life lost by premature death or disability, theses report do not estimate it directly for RSV. A valid and consistent description of the burden of disease is a great input to generate better health-policies and planning processes. Here, we estimated the disease burden of RSV infection in children less than 2 years in Colombia.

## Methods

### Design

Using the methods described by Murray and Lopez [5], we estimated the DALYs for RSV infection. DALYs were calculated for the most important health outcomes of this infection: RSV no complicated, RSV with or without acute mild or moderate complications (hypoxemia, atelectasis, pneumonia), RSV with severe acute complications (PICO admission, pneumothoraxes, pleural effusions, sepsis) and RSV infection with long term complications (recurrent wheezing). The study protocol was reviewed and approved by the Institutional Review Board of the University of Antioquia (No 18/2015).

### Model parameters and data sources

To estimate the burden of disease we use incidences and mortality rates from comprehensive data reported by the national report of epidemiological surveillance system during 2017 [7]. RSV is one of the notifiable diseases, it is mandatory for health providers, hospitals, and laboratories to report cases. The mortality data was validated with the data reported by the National Department of Statistics during the same time. Informed consent was not required because we used surveillance data without personal identifiers, Table 1.

**Table 1:** Model inputs: morbidity probabilities used in base case and sensitivity analyses

Model input	Base case value	SA range for one-way sensitivity analyses	Source
**Probability**			
Mortality by RSV in hospitalization	0,009	0,001-0,067	[7–12]
Mortality by RSV in pediatric intensive critical unit	0,036	0,021-0,052	
Incidence of acute complications in hospitalization	0,131	0,101–0,202	
Incidence of acute complications in pediatric intensive critical unit	0,153	0,150-0,536	
Probability of recurrent wheeze in RSV	0,285	0,237-0,289	
**Disability weight**			
Mild or moderate lower respiratory infections	0,051	0,032-0,074	[13]
Severe lower respiratory infections	0,133	0,008–0.19	
Recurrent wheeze	0,133	0,086-0,192	

*SA* sensitivity analyses

To estimate the ranges of incidence and mortality rates, systematic review of studies previously published with Colombian patients was made. This search was performed in February 2019 and was limited to published primary literature in the English or Spanish language, human subjects, and children (birth to 5 years). The following engines were searched for the periods specified: MEDLINE from 1950 on, EMBASE from 1974 on, BIREME from 1980 on. To avoid missing any articles published we performed a search using Google search engine, we reviewed the first 100 results returned of this search. Terms for these database searches included keywords closely matching the relevant medical field headings: respiratory syncytial virus, and respiratory syncytial pneumovirus. The authors (JAB, JPS) reviewed all potentially relevant references independently and selected relevant publications. The inclusion criteria were observational studies, systematic reviews that reported the incidence or frequencies of clinical outcomes of a patient younger than 2 years with RSV infection, which included the Colombian or Latin American population. Twenty-seven studies were obtained of which five were included [8–12].

### Statistical analysis

The years of life lost by premature mortality were estimated, per outcome, by multiplying the number of deaths due to this outcome -in children with RSV under 2 years - by the number of years of expected remaining life at the age of death according to reference life tables recommended the manual of GBD studies [5] .All estimates used the Colombian population in 2017 [14]. The YLD per outcome was obtained by multiplying the number of cases –per outcome in children under 2 years with RSV infection - by both: the average duration of this outcome obtained from the literature [15], and respective disability weight derived from the 2015 GBD study, Table 1. The internal consistency of each parameter was evaluated using the DISMOD II program [16] following the recommendations of manual for national studies of the WHO disease burden [5]. To estimate the confidence interval around YLD, YLL, and DALYs, 10,000 iterations were made using a Monte Carlo simulation.. The DALYs was expressed both in absolute value and per 1000 person-years. Multi-way probabilistic sensitivity analysis was made using the standardized regression coefficient method [17]. In this analysis was evaluated the percentage of change in the total estimate of DALYs, evaluating each of the variables within its range, (Table 1) with a discount rate of 0 and 5%.

## Results

**Fig. 1:**
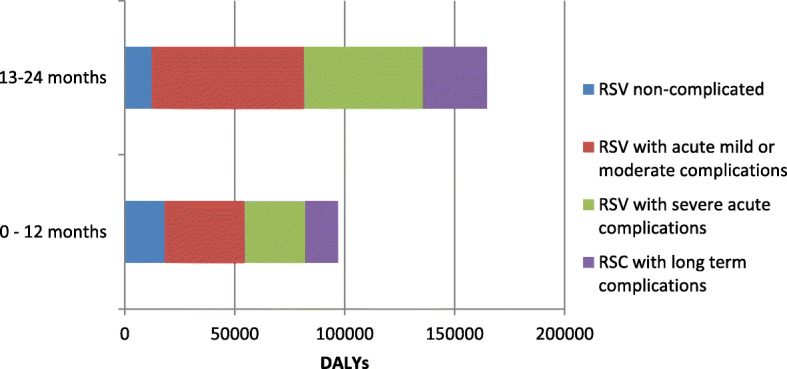
DALYs by outcome and age

In 2019, we estimated that ~ 260,873 years of life (CI 95% 208,180–347,023) were lost due to RSV infections in children under the age of 2 in Colombia. The estimated rate was 20 DALYs / 1000 person-year (95% CI 16–27). 51-% (1694 DALYs) were occurred in male children, and 63.19% of DALYs affected children between 1 to 2 years of age (Table 2). 99% of DALYs represented years of life lost due to early death. Around 40% of DALYs (104,632 DALYs) were generated by RSV with acute mild or moderate complications, followed by RSV with severe acute complications (31%), RSV infection with long term complications (16%) and uncomplicated RSV (12%), and this pattern was preserved in both age groups, Fig. 1.

**Table 2:** Distribution by sex and age of DALYs, YLL, YLD

	DALYS		YLD		YLL	
Age	Men	Female	Men	Female	Men	Female
**0–1 year**	49,037	46,789	270	258	48,766	46,531
**1–2 year**	84,083	80,386	579	553	83,504	79,833

Years of Adjusted Life by Disability [11], years of life lost due to premature death [1] and years of life lived with disability (YLD)

**Fig. 2:**
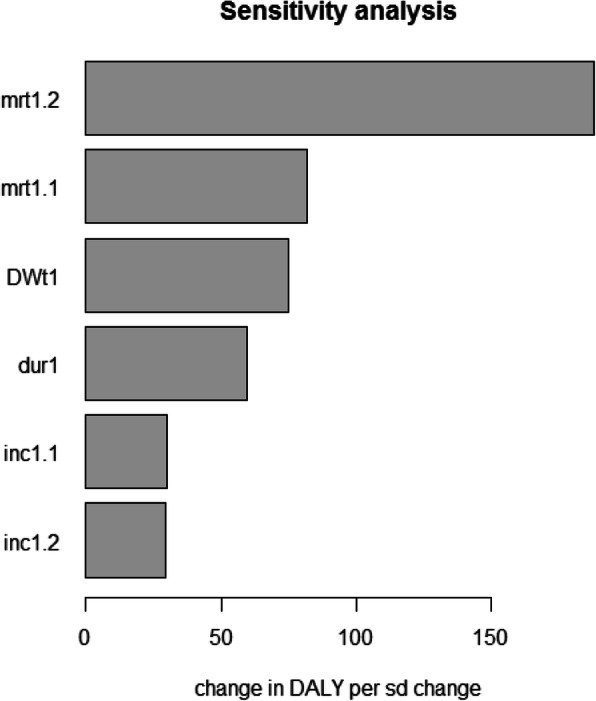
Results of sensitivity analysis: Mapped standardized regression coefficients

The results were robust in the sensitivity analysis. The percentage of change in the total estimate of DALYS did not exceed 25% with the variables analyzed; being the probability of death in children between 1 and 2 under the variable associated with the highest percentage of change in the DALYs (between 5 and 25%, of the final estimate). There were no significant variations in the discount rate, between 0 to 5% (Fig. 2).

## Discussion

This is, to our knowledge, the first estimate of burden of RSV in children under age 2 years in Colombia. We found that only RSV infection in children less than 2 years generates very significant number of years of life lost, highlighting not only the importance of this etiological agent but also the usefulness of using DALYs to assess the true weight of a disease in society. RSV is not the only risk factor, there are environmental and individual variables that condition the development of the infection. Estimating the impact of potentially modifiable factors such as RSV, allows to guide prioritization processes in intervention efficiently.

Respect to the difference to another burden of disease studies in our country, the global burden study estimated that LRTI accounts for around 14.1% of DALYs in children less than 4 years worldwide for example [13]. In Colombia, this percentage was 6.35% (9.9 DALYs per 1000 in children less than 4 years and 3.98 DALYs per 1000 in children less than 2 years) [4]. But our estimate is higher (was 20 DALYs / 1000 person-year (95% CI 16–27), and only included RSV. The national burden of disease study used data from national health surveys. Our study examined the records of epidemiological surveillance. These records have a greater degree of completeness since they are mandatory in Colombia, and are completed by the physicians and this tendency of a discrepancy of results has been seen before with other estimation of DALYs [18, 19].

Otherwise, if RSV infection alone generates 20 DALYs for 1000 children under 2 years of age, this disease would be only behind of low birth weight in the total estimation of DALYs in this age group. This finding is consistent with studies in the hospital setting where document the large amount of morbidity generated by RSV and acute bronchiolitis [1]. Most of DALYs (63.19%) were generated by children between 1 and 2 years, due to the greater amount of YLL lost, similar finding to other studies in Latin America [20]. It’s possible to explain this by a possible considerable delay in medical consultation of severe cases of RSV infection in older children, respect to younger children; due to attitude, more “relaxed” by parents with this patients. Further exist in older children a greater tendency to self-medication, aspects which affect the mortality rate [20].

When we compare our results with other estimations of DALYs of different diseases, RSV infection in children under age 2 years, generates more years of life lost than cervical cancer between 45 and 59 years (1.6 DALYs per 1000 inhabitants), epilepsy between 30 and 44 years (1 DALYs per 1000 inhabitants) and leukemia in children between 5 and 14 years (1 DALYs per 1000 inhabitants) [4]. This highlights the importance of generating specific burden of the disease studies by etiological agent, but also that it should encourage the development of vaccines, which according to our estimates would have a high population impact. Burden of disease studies should be a primary source for prioritization exercises in public health. Although in our continent even the use of health technology assessment and advanced statistical information is not the main input for decision-making, this type of estimations such as ours should encourage decision-makers to use evidence to make health decisions [21].

This study has limitations. First, we may have some degree of information bias and underestimation due to the use of data from the national surveillance and notification system [22]. However, LRTI cases have florid symptomatology in this age group, often prompting medical attention. There are a global increasing in the reporting of cases to SIVIGILA has been noted [7], and in our sensitivity analysis, the final result of DALYs was not sensitive to the change in values of these probabilities, guaranteeing the robustness of the model. There are no specific “disability weights” for RSV infection. In this case, we used data reported for LRTI because in terms of mortality it does not differ from data presented by patients with other viruses in Colombia [15]. In the sensitivity analysis, the percentage of change in the total estimate of DALYS did not exceed 25% within the variables analyzed.

## Conclusions

The burden of RSV bronchiolitis is a serious problem in Colombia, with a considerable social impact in terms of disability and mortality. Morbidity and mortality rates can be improved not only by effective prevention and promotion of public policies but also by improvements in the quality of health care services. Our results prompt evaluation of public health interventions and novel biological preventive strategies under evaluation to minimize the impact of this serious problem in Colombian children.

## Data Availability

The raw data supporting our findings can be requested from CIEMTO: http://ciemto.medicinaudea.co/contacto

## Abbreviations

YLD: Years of life lived with disability
SIVIGILA: National epidemiological surveillance system
GBD: Global Burden of Disease study

## References

[1] Nair H, Nokes DJ, Gessner BD, Dherani M, Madhi SA, Singleton RJ (2010). Global burden of acute lower respiratory infections due to respiratory syncytial virus in young children: a systematic review and meta-analysis. Lancet..

[2] Hall CB, Simoes EA, Anderson LJ (2013). Clinical and epidemiologic features of respiratory syncytial virus. Curr Top Microbiol Immunol.

[3] Sommer C, Resch B, Simoes EA (2011). Risk factors for severe respiratory syncytial virus lower respiratory tract infection. Open Microbiol J.

[4] Peñaloza R, Salamanca B, Rodriguez J, Beltran A. Estimación de la carga de enfermedad para Colombia, 2010. 2014(Editorial Pontificia Universidad Javeriana).

[5] Mathers CD VT, Lopez AD, Salomon J, Ezzati M. National Burden of Disease Studies: A Practical Guide: Global Program on Evidence for Health Policy. Geneva: World Health Organization.; 2001 [Available from: http://www.who.int/healthinfo/nationalburdenofdiseasemanual.pdf.

[6] Shi T, McAllister DA, O'Brien KL, Simoes EAF, Madhi SA, Gessner BD (2017). Global, regional, and national disease burden estimates of acute lower respiratory infections due to respiratory syncytial virus in young children in 2015: a systematic review and modelling study. Lancet..

[7] Instituto, Nacional, Salud d. Infeccion respiratoria aguda en Colombia 2017 [05/07/2019]. Available from: https://www.ins.gov.co/buscador-eventos/Informesdeevento/Informe%20IRA%20Final%202017.pdf. Accessed 5 Nov 2020.

[8] Buendia JA, Patino DG. Costs of Respiratory Syncytial Virus Hospitalizations in Colombia. Pharmacoecon Open. 2020. 10.1007/s41669-020-00218-7 PMC7895874 32418086

[9] Rodriguez-Martinez CE, Rodriguez DA, Nino G (2015). Respiratory syncytial virus, adenoviruses, and mixed acute lower respiratory infections in children in a developing country. J Med Virol.

[10] Rodriguez-Martinez CE, Sossa-Briceno MP, Castro-Rodriguez JA (2019). Cost-effectiveness of the utilization of "good practice" or the lack thereof according to a bronchiolitis evidence-based clinical practice guideline. J Eval Clin Pract.

[11] Rodriguez-Martinez CE, Sossa-Briceno MP, Castro-Rodriguez JA (2020). Direct medical costs of RSV-related bronchiolitis hospitalizations in a middle-income tropical country. Allergol Immunopathol (Madr).

[12] Pineros JG, Baquero H, Bastidas J, Garcia J, Ovalle O, Patino CM (2013). Respiratory syncytial virus infection as a cause of hospitalization in population under 1 year in Colombia. J Pediatr.

[13] Murray CJ, Barber RM, Foreman KJ, Abbasoglu Ozgoren A, DALYs GBD, Collaborators H (2015). Global, regional, and national disability-adjusted life years (DALYs) for 306 diseases and injuries and healthy life expectancy (HALE) for 188 countries, 1990-2013: quantifying the epidemiological transition. Lancet..

[14] Estadisticas DAN. Proyecciones de poblacion 2018 [03/07/2019]. Available from: https://www.dane.gov.co/index.php/estadisticas-por-tema/demografia-y-poblacion/proyecciones-de-poblacion. Accessed 5 Nov 2020.

[15] Barbosa Ramirez J, Pulido Dominguez P, Rey Benito G, Mendez Rico J, Castellanos J, Paez MA (2014). Human respiratory syncytial virus and metapneumovirus in patients with acute respiratory infection in Colombia, 2000–2011. Rev Panam Salud Publica.

[16] Barendregt JJ, Van Oortmarssen GJ, Vos T, Murray CJ (2003). A generic model for the assessment of disease epidemiology: the computational basis of DisMod II. Popul Health Metrics.

[17] Brecht Devleesschauwer, Scott McDonald, Juanita Haagsma, Nicolas Praet, Havelaar A, Speybroeck N. DALY: The DALY Calculator - A GUI for stochastic DALY calculation in R. 2014 Available from:. http://cran.rproject.org/package=DALY. Accessed 5 Nov 2020.

[18] Buendia JA, Chavarriaga GJR, Zuluaga AF (2019). Burden of paraquat poisoning in the department of Antioquia, Colombia. BMC Pharmacol Toxicol.

[19] Buendia JA, GJ R. Cost Ilness of paraquat poisoning in Colombia. Value Health Reg Issues. 2018;In Edition.

[20] Bardach A, Rey-Ares L, Cafferata ML, Cormick G, Romano M, Ruvinsky S (2014). Systematic review and meta-analysis of respiratory syncytial virus infection epidemiology in Latin America. Rev Med Virol.

[21] Pichon-Riviere A, Augustovski F, Garcia Marti S, Alfie V, Sampietro-Colom L. The link between health technology assessment and decision making for the allocation of health resources in Latin America. Int J Technol Assess Health Care. 2020;36(2):173–8. 10.1017/S0266462320000033 32312340

[22] Gibbons CL, Mangen MJ, Plass D, Havelaar AH, Brooke RJ, Kramarz P (2014). Measuring underreporting and under-ascertainment in infectious disease datasets: a comparison of methods. BMC Public Health.

